# Utilization of RNA *in situ* Hybridization to Understand the Cellular Localization of Powassan Virus RNA at the Tick-Virus-Host Interface

**DOI:** 10.3389/fcimb.2020.00172

**Published:** 2020-04-28

**Authors:** Meghan E. Hermance, Saravanan Thangamani

**Affiliations:** ^1^SUNY Center for Environmental Health and Medicine, SUNY Upstate Medical University, Syracuse, NY, United States; ^2^Institute for Global Health and Translational Science, SUNY Upstate Medical University, Syracuse, NY, United States; ^3^Department of Microbiology and Immunology, SUNY Upstate Medical University, Syracuse, NY, United States

**Keywords:** Powassan virus, tick-virus-host interface, skin, RNA *in situ* hybridization, tick feeding

## Abstract

Skin is the interface between an attached, feeding tick and a host; consequently, it is the first line of defense against invading pathogenic microorganisms that are delivered to a vertebrate host together with tick saliva. Central to the successful transmission of a tick-borne pathogen are complex interactions between the host immune response and early tick-mediated immunomodulation, all of which initially occur at the skin interface. The focus of this work was to demonstrate the use of RNA *in situ* hybridization (RNA ISH) as a tool for understanding the cellular localization of viral RNA at the feeding site of Powassan virus (POWV)-infected *Ixodes scapularis* ticks. Intense positive staining for POWV RNA was frequently detected in dermal foci and occasionally detected in hypodermal foci after 24 h of POWV-infected tick feeding. Additionally, duplex chromogenic RNA ISH staining demonstrated co-localization of POWV RNA with *Mus musculus* F4/80 RNA, CD11c RNA, vimentin RNA, Krt14 RNA, and CD3ε RNA at the feeding site of POWV-infected ticks. In future studies, RNA ISH can be used to validate transcriptomic analyses conducted at the tick-virus-host cutaneous interface and will provide cellular resolution for specific gene signatures temporally expressed during infected tick feeding. Such a systems biology approach will help create a more refined understanding of the cellular and molecular interactions influencing virus transmission at the cutaneous interface.

## Introduction

Mammalian skin serves as a mechanical and immunological barrier to protect the host from injury and infection (Pasparakis et al., 2014). This complex organ consists of an intricate network of epithelial cells, stromal cells, resident immune cells, migratory immune cells, blood and lymphatic vessels, peripheral nerves, and soluble mediators of the immune response (Wikel, 2013; Pasparakis et al., 2014; Nguyen and Soulika, 2019). Ticks are obligate blood-feeding ectoparasites of vertebrates and they require a blood meal at every active life stage. To acquire its necessary blood meal, an ixodid tick must remain attached to the skin of a vertebrate host and complete its multi-day feeding process without being deterred by the complex and redundant host cutaneous defense mechanisms. Successful tick feeding is enabled by the complex repertoire of bioactive factors in tick saliva, which can modulate host hemostasis, pain and itch responses, wound healing, and innate and adaptive immunity (Ribeiro et al., 2006; Kazimírová et al., 2017; Hermance and Thangamani, 2018; Wikel, 2018). Tick saliva is composed is of hundreds of proteins and short non-coding regulatory RNAs that are differentially expressed throughout the process of tick feeding (Ribeiro et al., 2006; Chmelar et al., 2016; Hermance et al., 2019). Studies have demonstrated that a variety of tick salivary proteins, as well as some non-proteinaceous molecules, can modulate the cutaneous immune defenses of the host (Ribeiro et al., 2006; Oliveira et al., 2011; Kazimírová et al., 2017).

Skin is the first mammalian organ that a tick-borne pathogen and tick salivary factors encounter during their journey from the infected tick salivary glands to the host. With respect to several tick-borne flaviviruses (TBFVs), an infected *Ixodes* species tick can transmit virus to the host on which it feeds in as little as a few minutes to several hours of feeding (Alekseev et al., 1996; Ebel and Kramer, 2004; Hermance and Thangamani, 2018). The rapid transmission of a TBFV from tick salivary glands to host underscores the importance of investigating the initial immunomodulatory events that occur at the cutaneous interface where a tick first delivers virus. *In vivo* models (infected ticks fed on small mammals) are valuable tools for studying the early host immune response to infected tick feeding. Using such models, transcriptional profiling and histopathological analyses conducted at the tick feeding site comprehensively illustrate how TBFV-infected tick feeding temporally influences the host cutaneous immune response. Comparative transcriptional analyses of TBFV-infected vs. uninfected tick feeding sites reveal significant up-regulation of transcripts related to the recruitment, migration, and accumulation of immune cells after the first 1–3 h of infected tick feeding (Hermance and Thangamani, 2014; Thangamani et al., 2017). Early cutaneous changes at the *in vivo* flavivirus-tick-host interface have been further illuminated with histopathological analyses. Until recently, these analyses have relied on immunohistochemical detection of viral antigen and specific cell markers at the tick feeding site. Specifically, Powassan virus (POWV) antigen was detected in fibroblasts and macrophages via immunofluorescence (Hermance et al., 2016), and Tick-borne encephalitis virus (TBEV) antigen was detected in fibroblasts and mononuclear phagocytes via immunohistochemistry (Thangamani et al., 2017). The present work is the first to demonstrate use of RNA *in situ* hybridization (RNA ISH) for precise localization of *Mus musculus*-specific F4/80, CD11c, vimentin, keratin 14, CD3ε, and CD49b RNAs at the tick feeding site, and to detect the distribution of POWV RNA at the skin site of POWV-infected *Ixodes scapularis* feeding. Simultaneous visualization POWV RNA with *Mus musculus*-specific mRNA targets is also demonstrated. Ultimately, a systems biology approach enables analyses of the complex interplay between tick-host-virus at the skin interface, and RNA ISH can be incorporated as a tool to visualize and validate cellular and molecular interactions occurring within the infected tick feeding site.

## Methods

### Cells and Viruses

African green monkey kidney (VeroE6) cells were purchased from the American Type Culture Collection (ATCC) and maintained in culture with Modified Eagle's Medium (MEM) supplemented with 10% fetal bovine serum (FBS), 1% non-essential amino acids, and a 1% antibiotic mixture of penicillin/streptomycin incubated at 37°C with 5% CO_2_. The World Reference Center for Emerging Viruses and Arboviruses at UTMB provided stock of the POWV (LB strain), which had previously been passaged 7 times in suckling mice brains. The stock was then passaged 5 times on VeroE6 cells. Stock virus titers were determined by focus-forming immunoassay as described previously (Rossi et al., 2012). Next Generation Sequencing demonstrated that the consensus nucleotide sequence of the POWV genome was 99.98% identical to the POWV LB strain. Two nucleotide sequence differences were observed between our POWV stock and that of the POWV LB strain (NCBI Reference Sequence: NC_003687.1), which resulted in the following amino acid sequence difference in: E (Q442R).

### Synchronous Infection of Ticks

POWV-infected *I. scapularis* nymphs were generated in the laboratory via synchronous infection, a technique that has been previously validated in our laboratory for virus infection of *I. scapularis* nymphs (Hermance and Thangamani, 2014; Hermance et al., 2016). Three days prior to synchronous infection, uninfected *I. scapularis* nymphs were stored in a 26°C environmental chamber at ~60% relative humidity for dehydration and desiccation. Ticks were then submerged in 1.5 × 10^7^ FFU/mL of POWV LB strain at 34°C for 45 min with gentle mixing every 10 min. Ticks were mock-infected in the same manner with DMEM media. Infected and mock-infected ticks were washed two times with sterile PBS and then dried of excess moisture using sterile filter paper. All ticks were transferred to glass vials and stored inside a desiccator for 4 weeks at 26°C and ~90% relative humidity to allow extrinsic incubation of POWV.

### Tick Infestation on Mice

Four weeks after synchronous infection, ticks were infested on mice as previously described (Hermance and Thangamani, 2014; Hermance et al., 2016). Briefly, tick containment capsules were fashioned from 2 mL cryotubes by cutting off the base of the tube, leaving approximately 5 mm of remaining tube and the screw cap lid intact. Tensoplast athletic tape (BSN Medical) was fitted around the perimeter of the base of the tube with approximately 1 cm of tape width around the edge of the capsule. Holes were punctured in the lid of the capsule with a 28-gauge needle to enable air-flow within the capsule during tick feeding. Mice were anesthetized with isoflurane for the capsule attachment process. The dorsum and lateral sides of each mouse was shaved with a size 40 electric razor blade. Livestock Tag Cement (Nasco) was applied to the adhesive side of the 1 cm athletic tape that was fitted around the edge of the capsule, and school glue was applied in a ring along the inner base of the capsule. Capsules were adhered to the center of the mouse dorsum 1 day prior to tick infestation. Capsule integrity was checked before and during the tick infestations.

One day after capsule attachment, a single *I. scapularis* nymph was placed inside each capsule. The screw-cap capsule lids were secured with a thin piece of masking tape. Ticks were fed on mice for 24 h. At 24 h after tick attachment (hours post-infection, hpi), mice were euthanized by CO_2_ inhalation followed by cervical dislocation. Immediately following euthanasia, 4 mm skin punch biopsies were harvested, including the attached ticks.

### Histology and RNA *in situ* Hybridization

Each skin plus tick biopsy was formalin-fixed for a minimum of 48 h in 10% neutral buffered formalin at room temperature. Tick legs were snipped prior to fixation to expedite formalin penetration into the tick body cavity. The biopsy samples were treated with Decal (StatLab) for 2 h at room temperature, thoroughly washed with DI water, and returned to formalin. The biopsies were then dehydrated with a standard ethanol series followed by xylene. Paraffin embedding of each biopsy was performed at an orientation that, upon sectioning, would produce a cross-section of the mouse skin and a longitudinal section of the tick mouthparts and body (Hermance et al., 2016). Formalin-fixed paraffin-embedded (FFPE) biopsies were sectioned at 5 μm thickness and sections were mounted on Superfrost Plus slides (Fisher Scientific). Slides were dried at 34°C for 48 h and then stored at room temperature in plastic bags with desiccants.

RNA ISH was performed with the RNAscope™ platform (Advanced Cell Diagnostics), including the RNAscope 2.5 Duplex reagent kit, RNAscope probes, and the RNAscope HybEZ hybridization system. RNA ISH was performed manually with a RNAscope 2.5 HD duplex chromogenic assay (Advanced Cell Diagnostics) in accordance with the manufacturer's recommendations for FFPE tissue. The FFPE tissue sections were incubated at 60°C for 1 h and deparaffinized in xylene. Endogenous peroxidases were quenched with H_2_O_2_ for 10 min at room temperature. Pretreatment times of the tissue sections were optimized so the delicate skin and brittle tick tissue would not lift off the slide during boiling or be damaged during protease digestion; therefore, slides underwent target retrieval for 15 min in RNAscope Target Retrieval Reagent at 98°C. Slides were then incubated for 15 min in RNAscope Protease Plus reagent at 40°C with constant humidity and temperature maintained by the RNAscope HybEZ hybridization system. RNA probes hybridizing to POWV positive-sense RNA (ACD Cat. # 415641), *Mus musculus* F4/80 RNA (Cat. # 460651-C2), *M. musculus* CD11c RNA (Cat. # 311501-C2), *M. musculus* vimentin RNA (Cat. # 457961-C2), *M. musculus* keratin 14 RNA (Cat. # 422521-C2), *M. musculus* CD3ε RNA (Cat. # 314721-C2), *M. musculus* CD49b RNA (Cat. # 441081-C2), positive control *M. musculus* ubiquitin C (Ubc) RNA (Cat. # 310771), positive control *M. musculus* Polr2a RNA (Cat. # 312471-C2), and positive control *M. musculus* Ppib RNA (Cat. # 321651) were hybridized to the tissues for 2 h at 40°C. All remaining steps of signal amplification and detection were conducted as recommended by the RNAscope 2.5 HD duplex detection kit user manual. Horseradish peroxidase (HRP)-based green and alkaline phosphatase (AP)-based red chromogenic substrates were used for signal detection. Slides were counterstained with Gill's Hematoxylin I (diluted 1:1 in DI water) for 30 s, briefly washed in tap water, air dried for 45 min at 60°C, and cover-slipped using VectaMount (Vector Laboratories) reagent. Tissue sections were examined under a standard light microscope.

## Results and Discussion

The RNAscope™ platform (Advanced Cell Diagnostics) used in this study reveals positive RNA signals as punctate staining, and previous work demonstrated that each punctate dot represents a single mRNA transcript (Wang et al., 2012). Probes targeting the endogenous *M. musculus* housekeeping genes, ubiquitin C (Ubc), Ppib, and Polr2a, were used as positive controls to assess mouse skin biopsies with attached ticks for RNA integrity and to serve as technical assay controls for the RNA ISH procedures. Endogenously expressed Ubc RNA was detected with HRP-based green chromogen in a punctate staining pattern and was moderately to highly expressed (>20 transcript copies per cell) in many *M. musculus* skin cells (Figure S1A). The Polr2a probe was used as a rigorous positive control for low copy mRNA (1–5 transcript copies per cell). Polr2a signal was detected with AP-based red chromogen, and as expected, was lowly expressed in *M. musculus* skin (Figures S1A,B). The Ppib housekeeping gene was used as a positive control for moderately expressed RNA targets (6–20 transcript copies per cell) (Figure S1B). The attached *I. scapularis* tick body in these tissue sections served as an internal negative control because the *M. musculus* Ubc, Polr2a, and Ppib probes did not bind to the tick tissue and only bound to the *M. musculus* skin, demonstrating probe specificity.

**Figure 1:**
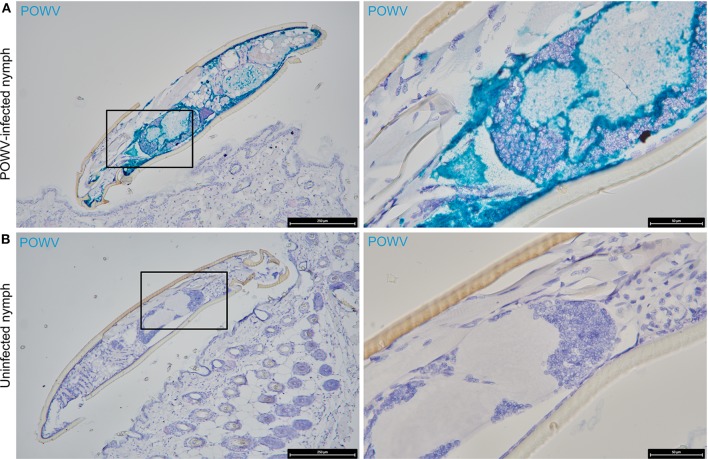
Distribution of POWV RNA in the POWV-infected *Ixodes scapularis* nymph body. **(A)** POWV-infected *I. scapularis* nymph feeding on *M. musculus* skin for 6 h. **(B)** Mock-infected *I. scapularis* nymph feeding on *M. musculus* skin for 6 h. POWV RNA is detected with HRP-based green chromogen and *M. musculus* F4/80 RNA is detected with AP-based red chromogen. For rows **(A,B)**, magnification is x10 and x40 from left to right. Black boxes on the left image panels indicate magnified regions shown in the right panels.

POWV RNA was detected via HRP-based green chromogen in the cross-section of POWV-infected *I. scapularis* nymphs but not in mock-infected nymphs (Figure 1). In the cross-sections of mock-infected nymphs, only the hematoxylin counterstain was visible (Figure 1B). In addition to the POWV RNA probe, the *M. musculus* F4/80 RNA probe was simultaneously hybridized to these skin plus nymph tissue sections, and duplex chromogenic detection of RNA targets was performed. No F4/80 RNA signal was detected via AP-based red chromogen in the tick bodies, demonstrating target-specificity of the *M. musculus* F4/80 RNA probe.

**Figure 2:**
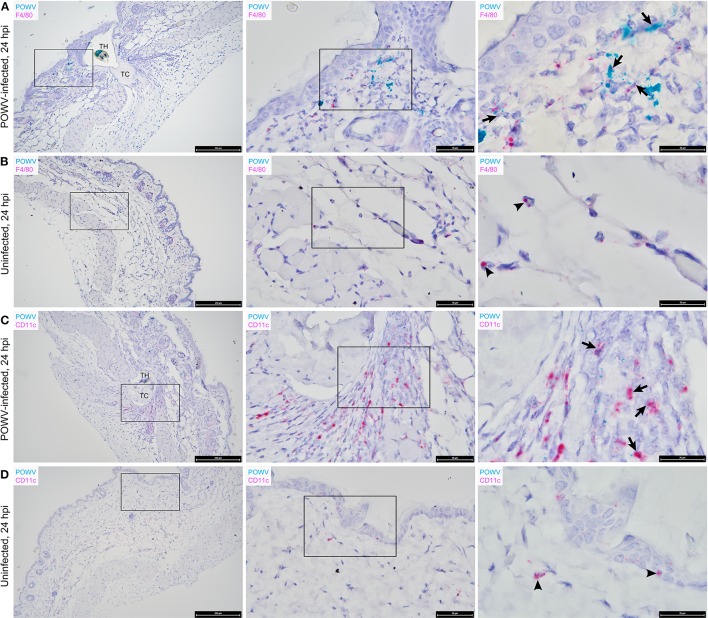
Distribution of POWV RNA, *Mus musculus* F4/80 RNA, and *M. musculus* CD11c RNA in the *Ixodes scapularis* nymph feeding site at 24 h post-tick attachment. **(A)** POWV-infected nymph feeding site showing POWV RNA (green signal) and *M. musculus* F4/80 RNA (red signal). Arrows indicate cells with co-localization of both RNA targets. **(B)** Mock-infected nymph feeding site with only *M. musculus* F4/80 RNA (red signal) detected. Arrowheads indicate cells with positive F4/80 RNA signal. **(C)** POWV-infected nymph feeding site showing POWV RNA (green signal) and *M. musculus* CD11c RNA (red signal). Arrows indicate cells with co-localization of both RNA targets. **(D)** Mock-infected nymph feeding site with only *M. musculus* CD11c RNA (red signal) detected. Arrowheads indicate cells with positive CD11c RNA signal. For rows **(A–D)**, magnification is x10, x40, x100 from left to right. Each black box indicates the magnified region shown in the adjacent right panel. TH, tick hypostome; TC, tick cement.

**Figure 3:**
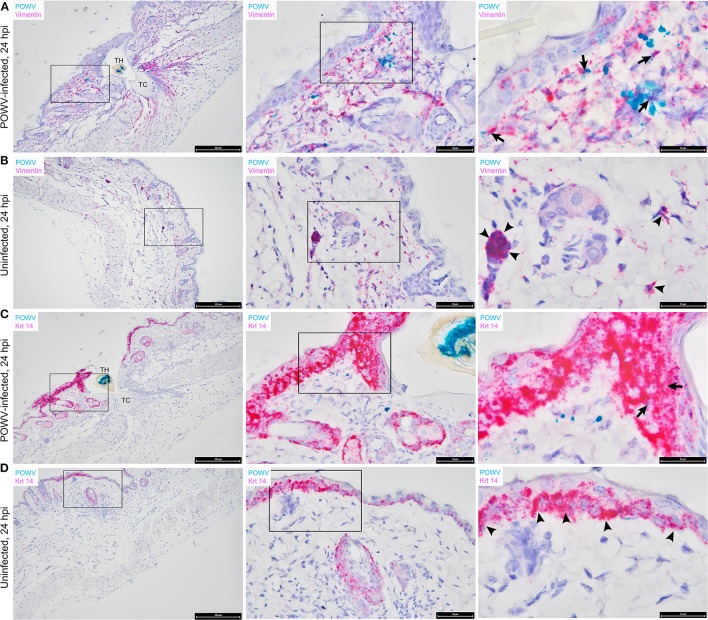
Distribution of POWV RNA, *Mus musculus* vimentin RNA, and *M. musculus* keratin 14 (Krt 14) RNA in the *Ixodes scapularis* nymph feeding site at 24 h post-tick attachment. **(A)** POWV-infected nymph feeding site showing POWV RNA (green signal) and *M. musculus* vimentin RNA (red signal). Arrows indicate cells with co-localization of both RNA targets. **(B)** Mock-infected nymph feeding site with only *M. musculus* vimentin RNA (red signal) detected. Arrowheads indicate cells with positive vimentin RNA signal. **(C)** POWV-infected nymph feeding site showing POWV RNA (green signal) and *M. musculus* Krt 14 RNA (red signal). Arrows indicate cells with co-localization of both RNA targets. **(D)** Mock-infected nymph feeding site with only *M. musculus* Krt 14 RNA (red signal) detected. Arrowheads indicate cells with positive Krt 14 RNA signal. For rows **(A–D)**, magnification is x10, x40, x100 from left to right. Each black box indicates the magnified region shown in the adjacent right panel. TH, tick hypostome; TC, tick cement.

**Figure 4:**
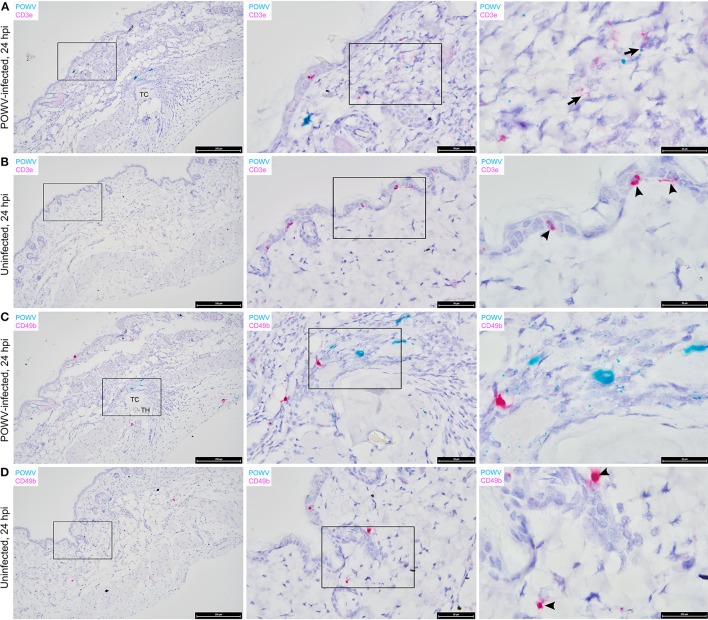
Distribution of POWV RNA, *Mus musculus* CD3ε RNA, and *M. musculus* CD49b RNA in the *Ixodes scapularis* nymph feeding site at 24 h post-tick attachment. **(A)** POWV-infected nymph feeding site showing POWV RNA (green signal) and *M. musculus* CD3ε RNA (red signal). Arrows indicate cells with co-localization of both RNA targets. **(B)** Mock-infected nymph feeding site with only *M. musculus* CD3ε RNA (red signal) detected. Arrowheads indicate cells with positive CD3ε RNA signal. **(C)** POWV-infected nymph feeding site showing POWV RNA (green signal) and *M. musculus* CD49b (red signal). No arrows are present because POWV RNA and CD49b RNA signals did not co-localize in this tick feeding site. **(D)** Mock-infected nymph feeding site with only *M. musculus* CD49b (red signal) detected. Arrowheads indicate cells with positive CD3ε RNA signal. For rows **(A–D)**, magnification is x10, x40, x100 from left to right. Each black box indicates the magnified region shown in the adjacent right panel. TH, tick hypostome; TC, tick cement.

The main focus of this work was to demonstrate the use of RNA ISH technology as a tool for detecting the distribution of POWV RNA at the cutaneous feeding site of an infected tick while identifying co-localization of the POWV RNA signal with secondary mammalian-specific RNA targets of interest. In this study, POWV RNA was routinely detected with HRP-based green chromogen at the feeding site of POWV-infected *I. scapularis* nymphs that were fed on mice for 24 h (Figures 2–4). Intense viral RNA staining was often focally detected in the dermis (Figures 2A,C, 3A,C, 4A,C), and in some sections, intense POWV RNA signal was also detected in hypodermal foci (Figures 4A,C). Positive staining for POWV RNA in the epidermis was less common and weaker overall than the POWV RNA signals in the dermis (Figures 2A, 3A,C). The epidermis is the thinnest layer of murine skin, and nymphal tick mouthparts penetrate well-beyond the epidermis, reaching the dermal, and hypodermal layers of skin; therefore, it is not surprising that we detected less POWV RNA in the epidermis than the dermis. For skin cross-sections that include portions of POWV-infected tick mouthparts, POWV RNA was frequently identified within the bounds of the tick mouthparts (Figures 2A,C, 3A,C).

We previously used the F4/80 antigen as an immunohistochemical marker for macrophages at the skin feeding site of POWV-infected ticks and demonstrated that macrophages contained POWV antigen (Hermance et al., 2016). In this study, we sought to detect F4/80 transcript expression within tissue context during POWV-infected tick feeding. POWV RNA and *M. musculus* F4/80 RNA probes were hybridized to the POWV-infected and mock-infected *I. scapularis* nymph feeding sites (Figures 2A,B). F4/80 RNA was detected with AP-based red chromogen in both the POWV-infected and mock-infected nymph feeding sites, and the F4/80 RNA signal was scattered throughout the dermis and hypodermis of these sections. Additionally, in the POWV-infected nymph feeding site, multiple F4/80-positive RNA signals were detected in the hypodermis and sub-muscular layer adjacent to the tick cement deposits and were likely expressed by the mononuclear cells infiltrating the tick feeding site (Figure 2A). Co-localization of POWV RNA with F4/80 RNA was detected in in the dermis of the POWV-infected tick feeding site (Figure 2A); however, as expected, co-localization of POWV and F4/80 RNA signals was not detected in the mock-infected tick feeding site (Figure 2B).

The distribution of POWV RNA and *M. musculus* CD11c RNA in the tick feeding sites was detected via HRP-based green chromogen and AP-based red chromogen, respectively (Figures 2C,D). CD11c RNA signals were detected in the epidermis, dermis, hypodermis, and sub-muscular layer of the *I. scapularis* feeding sites. Epidermal cells with CD11c-positive RNA signal are likely Langerhans cells, which are the main dendritic cell subpopulation in the epidermis (Kazimírová et al., 2017). In the present study, cells in which POWV RNA co-localized with CD11c RNA were located in the hypodermal and sub-muscular regions adjacent to the cement deposited by the feeding POWV-infected nymph (Figure 2C). These compartments of the skin included multiple cells with CD11c-positive RNA signal, which can likely be attributed to mononuclear phagocyte infiltrates recruited to the *I. scapularis* feeding site. Although we did not detect co-localization of POWV RNA with CD11c RNA in epidermal cells, our findings do not preclude Langerhans cells as putative cell targets of POWV infection at the tick feeding site. A previous study detected TBEV antigen in Langerhans cells emigrating from skin explants harvested at the feeding site of TBEV-infected ticks, suggesting that Langerhans cells are early cutaneous cell targets of TBEV infection and that they serve as vehicles for virus dissemination to skin-draining lymph nodes (Labuda et al., 1996). Furthermore, Langerhans cell migration to draining lymph nodes has been demonstrated in response to cutaneous infection with live West Nile virus (Johnston et al., 2000). Therefore, it is possible that some of the hypodermal and sub-muscular cells in this study that displayed co-localized POWV and CD11c RNA signals were Langerhans cells or dermal dendritic cells migrating from the tick feeding site to the lymphatic system.

Vimentin transcript expression was characterized *in situ* in the presence of POWV-infected vs. uninfected *I. scapularis* nymph feeding. *M. musculus* vimentin RNA was detected throughout the epidermis, dermis, hypodermis, and sub-muscular layers of the skin (Figures 3A,B). The vimentin gene encodes a type III intermediate filament protein which participates in numerous cellular functions, including cell adhesion, migration, differentiation, cytoskeletal rearrangements, and wound healing (Cheng et al., 2016). The most intense staining for vimentin RNA was detected near the feeding lesion of the tick where the tissue architecture, including the sub-muscular layer, had the appearance of streaming toward the tick mouthparts (Figures 3A,B). Previous studies conducted at the 24-h feeding site of uninfected and POWV-infected *I. scapularis* nymphs demonstrated upregulated gene ontology clusters related to cytoskeletal arrangements and histopathological changes related to wound healing (Heinze et al., 2012; Hermance et al., 2016); therefore, increased vimentin transcript expression in sites adjacent to the tick feeding lesion after 24 h of tick feeding was anticipated in these samples. Furthermore, RNA ISH revealed co-localization of POWV RNA with vimentin RNA (Figure 3A), supporting previous immunohistochemical findings where POWV and vimentin antigens co-localized at the POWV-infected tick feeding site (Hermance et al., 2016).

The distribution of *M. musculus* Keratin 14 (Krt14) RNA was characterized at the tick feeding site via RNA ISH. Krt14 is expressed in the basal layer of the epidermis and in hair follicles, and it is a prototypic marker of proliferative basal keratinocytes (Coulombe et al., 1989; Alam et al., 2011; Wang et al., 2016). The mouse skin biopsies included in this study displayed Krt14-positive RNA signals in the epidermis and hair follicles (Figures 3C,D). Here, several punctate dots of POWV RNA signal co-localized with Krt14 RNA in the epidermis of the POWV-infected tick feeding site (Figure 3C). This suggests that keratinocytes are early cutaneous cell targets of POWV infection at the tick feeding site. Previous reports have implicated keratinocytes as initial cell targets of flavivirus infection *in vivo*, which supports our findings (Labuda et al., 1996; Lim et al., 2011; Hamel et al., 2015). Additionally, *M. musculus* CD3ε RNA and CD49b RNA were also detected in the POWV-infected and mock-infected nymph feeding sites (Figure 4). The CD3ε-positive RNA staining was detected focally in the epidermis and dermis (Figures 4A,B), while foci of CD49b RNA were identified in all layers of the skin biopsies taken at the tick feeding sites (Figures 4C,D). POWV RNA co-localized with CD3ε RNA but not with CD49b RNA at the 24-h feeding site of the POWV-infected *I. scapularis* nymph (Figure 4A).

Identification of mammalian immune cells that are early targets of TBFV infection at the cutaneous interface contributes to our overall understanding of virus pathogenesis in the mammalian host. The present study adds knowledge to this realm via RNA ISH detection of POWV RNA and mammalian-specific RNAs at the tick feeding site, and the findings are summarized in Table 1. Although co-localization of POWV RNA and mammalian RNA targets suggests that a cell is infected with POWV, this does not confirm whether viral proteins co-localize with the mammal-specific protein or that the mammalian-specific transcript is involved in the lifecycle of POWV. A limitation of this study is that the RNA signals were not quantified. The development of a digital analysis program that could quantify individual RNA signals, as well as the relative degree of signal co-localization between two transcripts, would be an asset for future studies of this nature. Furthermore, this study detected POWV lineage I RNA at the feeding site of *I. scapularis* nymphs; however, results could vary for ticks infected with POWV lineage II, otherwise known as deer tick virus. Viral RNA distribution patterns at the cutaneous feeding site of a deer tick virus-infected tick could be different from that of a POWV-infected tick. Future studies investigating differences in the viral RNA distribution between both virus genotypes would be beneficial.

**Table 1:** Summary of RNA ISH findings at the POWV-infected *Ixodes scapularis* nymph feeding site.

***M. musculus* target transcript**	**Location of target transcript in the skin**	***M. musculus* cells expressing this target transcript**	**Co-localization of target transcript with POWV RNA?**
F4/80	Dermis, hypodermis, sub-muscular layer	Macrophages, monocytes	Yes
CD11c	Epidermis, dermis, hypodermis, sub-muscular layer	Dendritic cells, Langerhans cells, macrophages, NK cells	Yes
Vimentin	Epidermis, dermis, hypodermis, sub-muscular layer	Mesenchymal cells	Yes
Keratin 14	Epidermis, hair follicles	Keratinocytes	Yes
CD3ε	Epidermis, dermis	T cells	Yes
CD49b	Epidermis, dermis, hypodermis, sub-muscular layer	Lymphocytes	No

## Conclusion

In summary, the present study is the first to demonstrate the use of RNA ISH technology for precise cellular localization of viral RNA at the tick feeding site. This technology can be applied to studies with other vector-borne pathogens to investigate cellular localization of pathogen RNA and the potential immune responses occurring at the vector-pathogen-host interface. Here, we detected the distribution of POWV RNA at the feeding site of POWV-infected *I. scapularis* nymphs and detected co-localization of POWV RNA with *M. musculus* F4/80 RNA, CD11c RNA, vimentin RNA, Krt14 RNA, and CD3ε RNA. In future studies, RNA ISH can serve as a validation tool for transcriptomic analyses investigating the host cutaneous immune response during infected tick feeding. This technology will also enable us to map specific gene expression signals to individual cells or compartments within the infected tick feeding site. Using RNA ISH to acquire cellular resolution for specific gene expression patterns will improve our understanding of the cellular and molecular interactions occurring at the tick-virus-host interface.

## Data Availability Statement

All datasets generated for this study are included in the article/Supplementary Material.

## Ethics Statement

All experiments involving mice were conducted in arthropod containment biosafety level 3 (ACL-3) facilities in strict accordance with an animal use protocol approved by the University of Texas Medical Branch (UTMB) Institutional Animal Care and Use Committee (IACUC: # 0907054).

## Author Contributions

MH and ST designed the experiments and analyzed the data. ST provided reagents and materials, and critically read and revised the manuscript. MH performed the experiments and drafted the manuscript.

## Conflict of Interest

The authors declare that the research was conducted in the absence of any commercial or financial relationships that could be construed as a potential conflict of interest.

## Supplementary Material

Figure S1 Positive controls for RNA ISH of skin sections. **(A)** *I. scapularis* nymph feeding site where *M. musculus* ubiquitin (Ubc) RNA is detected with an HRP-based green chromogen and Polr2a RNA is detected with an AP-based red signal. **(B)** *I. scapularis* nymph feeding site where *M. musculus* Ppib RNA is detected with an HRP-based green chromogen and Polr2a RNA is detected with an AP-based red signal. For rows **(A,B)**, magnification is x10, x40 from left to right. Black boxes on the left image panels indicate magnified regions shown in the right panels.

## References

[B1] Alam H. Sehgal L. Kundu S. T. Dalal S. N. Vaidya M. M. (2011). Novel function of keratins 5 and 14 in proliferation and differentiation of stratified epithelial cells. Mol. Biol. Cell. 22, 4068–4078. 10.1091/mbc.E10-08-0703 21900500 PMC3204069

[B2] Alekseev A. N. Burenkova L. A. Vasilieva I. S. Dubinina H. V. Chunikhin S. P. (1996). Preliminary studies on virus and spirochete accumulation in the cement plug of ixodid ticks. Exp. Appl. Acarol. 20, 713–723. 10.1007/bf00051556 9004495

[B3] Cheng F. Shen Y. Mohanasundaram P. Lindström M. Ivaska J. Ny T.. (2016). Vimentin coordinates fibroblast proliferation and keratinocyte differentiation in wound healing via TGF-β-Slug signaling. Proc. Natl. Acad. Sci. U.S.A. 113, e4320–e4327. 10.1073/pnas.1519197113 27466403 PMC4968728

[B4] Chmelar J. Kotál J. Karim S. Kopacek P. Francischetti I. M. B. Pedra J. H. F.. (2016). Sialomes and mialomes: a systems-biology view of tick tissues and tick-host interactions. Trends Parasitol. 32, 242–254. 10.1016/j.pt.2015.10.002 26520005 PMC4767689

[B5] Coulombe P. A. Kopan R. Fuchs E. (1989). Expression of keratin K14 in the epidermis and hair follicle: insights into complex programs of differentiation. J. Cell Biol. 109, 2295–2312. 10.1083/jcb.109.5.2295 2478566 PMC2115845

[B6] Ebel G. D. Kramer L. D. (2004). Short report: duration of tick attachment required for transmission of powassan virus by deer ticks. Am. J. Trop. Med. Hyg. 71, 268–271. 10.4269/ajtmh.2004.71.3.0700268 15381804

[B7] Hamel R. Dejarnac O. Wichit S. Ekchariyawat P. Neyret A. Luplertlop N.. (2015). Biology of zika virus infection in human skin cells. J. Virol. 89, 8880–8896. 10.1128/JVI.00354-15 26085147 PMC4524089

[B8] Heinze D. M. Carmical J. R. Aronson J. F. Thangamani S. (2012). Early immunologic events at the tick-host interface. PLoS ONE 7:e47301. 10.1371/journal.pone.0047301 23077588 PMC3471850

[B9] Hermance M. E. Santos R. I. Kelly B. C. Valbuena G. Thangamani S. (2016). Immune cell targets of infection at the tick-skin interface during powassan virus transmission. PLoS ONE 11:e0155889. 10.1371/journal.pone.0155889 27203436 PMC4874601

[B10] Hermance M. E. Thangamani S. (2014). Proinflammatory cytokines and chemokines at the skin interface during Powassan virus transmission. J. Invest. Dermatol. 134, 2280–2283. 10.1038/jid.2014.150 24658509 PMC4102615

[B11] Hermance M. E. Thangamani S. (2018). Tick-virus-host interactions at the cutaneous interface: the nidus of flavivirus transmission. Viruses 10:362 10.3390/v10070362 PMC6071252 29986483

[B12] Hermance M. E. Widen S. G. Wood T. G. Thangamani S. (2019). Ixodes scapularis salivary gland microRNAs are differentially expressed during Powassan virus transmission. Sci. Rep. 9:13110. 10.1038/s41598-019-49572-5 31511580 PMC6739385

[B13] Johnston L. J. Halliday G. M. King N. J. (2000). Langerhans cells migrate to local lymph nodes following cutaneous infection with an arbovirus. J. Invest. Dermatol. 114, 560–568. 10.1046/j.1523-1747.2000.00904.x 10692118

[B14] Kazimírová M. Thangamani S. Bartíková P. Hermance M. Holíková V. Štibrániová I.. (2017). Tick-borne viruses and biological processes at the tick-host-virus interface. Front. Cell. Infect. Microbiol. 7:339. 10.3389/fcimb.2017.00339 28798904 PMC5526847

[B15] Labuda M. Austyn J. M. Zuffova E. Kozuch O. Fuchsberger N. Lysy J.. (1996). Importance of localized skin infection in tick-borne encephalitis virus transmission. Virology. 219, 357–366. 10.1006/viro.1996.0261 8638401

[B16] Lim P. Y. Behr M. J. Chadwick C. M. Shi P. Y. Bernard K. A. (2011). Keratinocytes are cell targets of West Nile virus *in vivo*. J. Virol. 85, 5197–5201. 10.1128/JVI.02692-10 21367890 PMC3126165

[B17] Nguyen A. V. Soulika A. M. (2019). The dynamics of the skin's immune system. Int. J. Mol. Sci. 20:e1811. 10.3390/ijms20081811 31013709 PMC6515324

[B18] Oliveira C. J. Sá-Nunes A. Francischetti I. M. Carregaro V. Anatriello E. Silva J. S.. (2011). Deconstructing tick saliva: non-protein molecules with potent immunomodulatory properties. J. Biol. Chem. 286, 10960–10969. 10.1074/jbc.M110.205047 21270122 PMC3064151

[B19] Pasparakis M. Haase I. Nestle F. O. (2014). Mechanisms regulating skin immunity and inflammation. Nat. Rev. Immunol. 14, 289–301. 10.1038/nri3646 24722477

[B20] Ribeiro J. M. Alarcon-Chaidez F. Francischetti I. M. Mans B. J. Mather T. N. Valenzuela J. G.. (2006). An annotated catalog of salivary gland transcripts from ixodes scapularis ticks. Insect. Biochem. Mol. Biol. 36, 111–129. 10.1016/j.ibmb.2005.11.005 16431279

[B21] Rossi S. L. Nasar F. Cardosa J. Mayer S. V. Tesh R. B. Hanley K. A.. (2012). Genetic and phenotypic characterization of sylvatic dengue virus type 4 strains. Virology 423, 58–67. 10.1016/j.virol.2011.11.018 22178263 PMC3253985

[B22] Thangamani S. Hermance M. E. Santos R. I. Slovak M. Heinze D. Widen S. G.. (2017). Transcriptional immunoprofiling at the tick-virus-host interface during early stages of tick-borne encephalitis virus transmission. Front. Cell. Infect. Microbiol. 7:494. 10.3389/fcimb.2017.00494 29250492 PMC5716978

[B23] Wang F. Flanagan J. Su N. Wang L. C. Bui S. Nielson A.. (2012). RNAscope: a novel *in situ* RNA analysis platform for formalin-fixed, paraffin-embedded tissues. J. Mol. Diagn. 14, 22–29. 10.1016/j.jmoldx.2011.08.002 22166544 PMC3338343

[B24] Wang F. Zieman A. Coulombe P. A. (2016). Skin keratins. Methods Enzymol. 568, 303–350. 10.1016/bs.mie.2015.09.032 26795476 PMC4902878

[B25] Wikel S. K. (2013). Ticks and tick-borne pathogens at the cutaneous interface: host defenses, tick countermeasures, and a suitable environment for pathogen establishment. Front. Microbiol. 4:337. 10.3389/fmicb.2013.00337 24312085 PMC3833115

[B26] Wikel S. K. (2018). Tick-host-pathogen systems immunobiology: an interactive trio. Front. Biosci. 23, 265–283. 10.2741/4590 28930546

