# Supplementary figures and images for: Utilization of RNA in situ Hybridization to Understand the Cellular Localization of Powassan Virus RNA at the Tick-Virus-Host Interface
Source: Front Cell Infect Microbiol. 2020 Apr 28;10:172. doi: 10.3389/fcimb.2020.00172 (PMC7198705; doi:10.3389/fcimb.2020.00172)

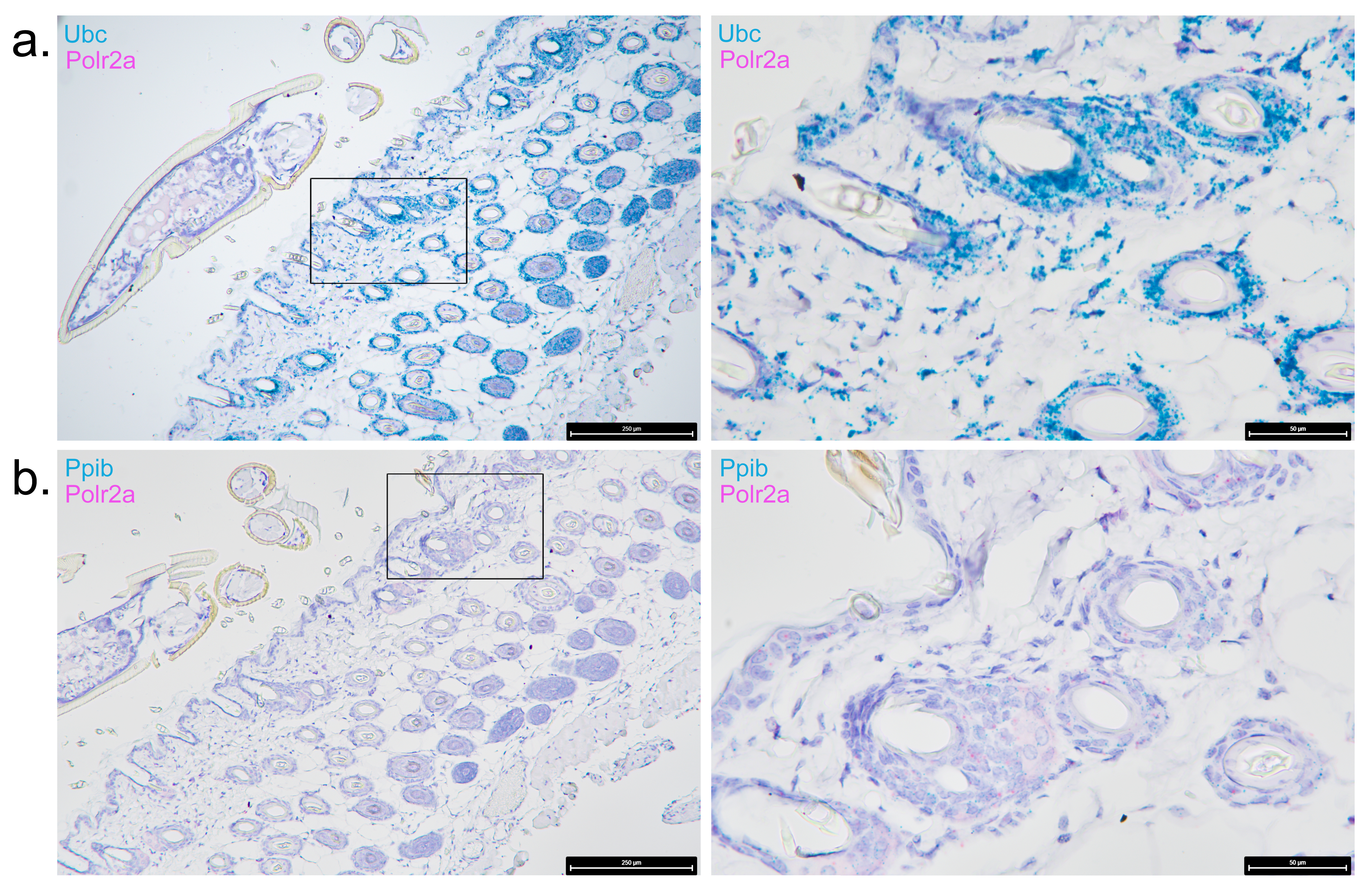

Supplement: Figure S1 — Positive controls for RNA ISH of skin sections. (A) I. scapularis nymph feeding site where M. musculus ubiquitin (Ubc) RNA is detected with an HRP-based green chromogen and Polr2a RNA is detected with an AP-based red signal. (B) I. scapularis nymph feeding site where M. musculus Ppib RNA is detected with an HRP-based green chromogen and Polr2a RNA is detected with an AP-based red signal. For rows (A,B), magnification is x10, x40 from left to right. Black boxes on the left image panels indicate magnified regions shown in the right panels. [file Image_1.JPEG]
